# 3,3′-Bithio­phene

**DOI:** 10.1107/S1600536810010342

**Published:** 2010-03-24

**Authors:** José C. S. Costa, Ligia R. Gomes, Luís M. N. B. F. Santos, John Nicolson Low

**Affiliations:** a Centro de Investigação em Química, Departamento de Química e Bioquímica, Faculdade de Ciências, Universidade do Porto, Rua do Campo Alegre, 687, P-4169 007 Porto, Portugal; b REQUIMTE , Departamento de Química e Bioquímica, Faculdade de Ciências, Universidade do Porto, Rua do Campo Alegre, 687, P-4169 007 Porto, Portugal; c Department of Chemistry, University of Aberdeen, Meston Walk, Old Aberdeen AB24 3UE, Scotland

## Abstract

The title compound, C_8_H_6_S_2_, is disordered [occupancy ratio =  0.839 (2):0.161 (2)] and sits across a centre of symmetry. In the crystal, the mol­ecules are linked by a weak C—H⋯π inter­action.

## Related literature

For a discussion of the disorder in this compound, see: Visser *et al.* (1968 ▶). For thio­phene C–S bond distances, see: Allen *et al.* (1987 ▶).
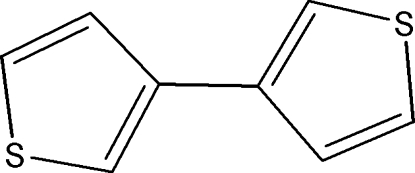


## Experimental

### 

#### Crystal data

C_8_H_6_S_2_
*M* *_r_* = 166.25
Orthorhombic, 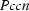

*a* = 7.5187 (7) Å
*b* = 18.2181 (17) Å
*c* = 5.5029 (5) Å
*V* = 753.77 (12) Å^3^
*Z* = 4
Mo *K*α radiation
μ = 0.62 mm^−1^
*T* = 150 K
0.60 × 0.40 × 0.04 mm

#### Data collection

Bruker SMART APEXII diffractometer
Absorption correction: multi-scan (*SADABS*; Bruker, 2004 ▶) *T*_min_ = 0.709, *T*_max_ = 0.976
11635 measured reflections
1151 independent reflections
987 reflections with *I* > 2σ(*I*)
*R*_int_ = 0.039

#### Refinement

*R*[*F* ^2^ > 2σ(*F* ^2^)] = 0.039
*wR*(*F* ^2^) = 0.101
*S* = 1.10
1151 reflections
59 parameters
6 restraints
H-atom parameters constrained
Δρ_max_ = 0.48 e Å^−3^
Δρ_min_ = −0.33 e Å^−3^

### 

Data collection: *APEX2* (Bruker, 2004 ▶); cell refinement: *SAINT* (Bruker, 2004 ▶); data reduction: *SAINT*; program(s) used to solve structure: *SHELXS97* (Sheldrick, 2008 ▶); program(s) used to refine structure: *SHELXL97* (Sheldrick, 2008 ▶); molecular graphics: *ORTEPII* (Johnson, 1976 ▶) and *PLATON* (Spek, 2009 ▶); software used to prepare material for publication: *SHELXL97*.

## Supplementary Material

Crystal structure: contains datablocks global, I. DOI: 10.1107/S1600536810010342/om2327sup1.cif

Structure factors: contains datablocks I. DOI: 10.1107/S1600536810010342/om2327Isup2.hkl

Additional supplementary materials:  crystallographic information; 3D view; checkCIF report

## Figures and Tables

**Table 1:** Hydrogen-bond geometry (Å, °) *Cg* and *Cg*′ are the centroids of the thio­phene ring in the major and minor occupancy disorder components, respectively.

*D*—H⋯*A*	*D*—H	H⋯*A*	*D*⋯*A*	*D*—H⋯*A*
C2—H2⋯*Cg*^i^	0.95	2.86	3.6039 (17)	136
C2—H2⋯*Cg*′^i^	0.95	2.86	3.607 (5)	136

Symmetry code: (i) 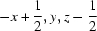.

**Fig. 1.**
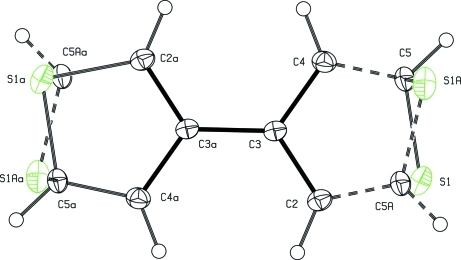
A view of the title compound with our numbering scheme. Displacement ellipsoids are drawn at the 30% probability level. The molecule sits across the centre-of-symmetry at (0.5,0.5,0.5). The bonds in the minor component are marked as dotted lines.

## supplementary crystallographic information

### Comment

The disorder in the title compound was discussed briefly by Visser *et al.* (1968). However, this paper gives no coordinates and the structure determination was at room temperature. This is a low temerature determination. A view of the major, 0.839 (2), site occupancy, and minor, 0.161 (2), site occupancy, components are shown in Fig. 1. There is a weak C–H···π interaction, C2–H2···Cg(thiophene) (0.5-x, y, z-0.5) in which H2···Cg is 2.86Å and C2···Cg is 3.6039 (17) Å. The angle at H2 ia 136° for the major component. The C2···Cg2 distance for the minor component is 3.607 (5) Å. The H2···Cg distance and angle at H2 are the same.

### Experimental

The compound was obtained commercially and re-crystallised from dichloromethane.

### Refinement

H atoms were treated as riding atoms with C—H(aromatic), 0.95Å. The S atom was disordered by rotation of 180° around the bond connecting the 2 thiophene rings. The C—S distances were restrained the average value quoted in Allen, *et al.*, 1987 using tight restraints. Specifically, the C2-C5a and C4-C5 bonds were restrained in SHELXL97 refinements using DFIX 1.380 0.001 and the C5-S1, C2-S1, C4-S1A and C5A-S1A bonds were restrained using DFIX 1.72 0.001. The anisotropic thermal parameters for atom C5A (minor component) were constrained to be the same as those those of atom C5 (major component) using the EADP instruction.

### Crystal data

**Table:**

C_8_H_6_S_2_	*D*_x_ = 1.465 Mg m^−^^3^
*M**_r_* = 166.25	Melting point: 406 K
Orthorhombic, *P**c**c**n*	Mo *K*α radiation, λ = 0.71073 Å
*a* = 7.5187 (7) Å	Cell parameters from 124 reflections
*b* = 18.2181 (17) Å	θ = 2.8–30.5°
*c* = 5.5029 (5) Å	µ = 0.62 mm^−^^1^
*V* = 753.77 (12) Å^3^	*T* = 150 K
*Z* = 4	Plate, yellow
*F*(000) = 344	0.60 × 0.40 × 0.04 mm

### Data collection

**Table:**

Bruker SMART APEXII diffractometer	1151 independent reflections
Radiation source: fine-focus sealed tube	987 reflections with *I* > 2σ(*I*)
graphite	*R*_int_ = 0.039
ω scans	θ_max_ = 30.6°, θ_min_ = 4.3°
Absorption correction: multi-scan (*SADABS*; Bruker, 2004)	*h* = −10→10
*T*_min_ = 0.709, *T*_max_ = 0.976	*k* = −23→26
11635 measured reflections	*l* = −7→7

### Refinement

**Table:**

Refinement on *F*^2^	Primary atom site location: structure-invariant direct methods
Least-squares matrix: full	Secondary atom site location: difference Fourier map
*R*[*F*^2^ > 2σ(*F*^2^)] = 0.039	Hydrogen site location: inferred from neighbouring sites
*wR*(*F*^2^) = 0.101	H-atom parameters constrained
*S* = 1.10	*w* = 1/[σ^2^(*F*_o_^2^) + (0.0478*P*)^2^ + 0.3736*P*] where *P* = (*F*_o_^2^ + 2*F*_c_^2^)/3
1151 reflections	(Δ/σ)_max_ = 0.001
59 parameters	Δρ_max_ = 0.48 e Å^−^^3^
6 restraints	Δρ_min_ = −0.33 e Å^−^^3^

### Special details

**Table:**

Geometry. All esds (except the esd in the dihedral angle between two l.s. planes) are estimated using the full covariance matrix. The cell esds are taken into account individually in the estimation of esds in distances, angles and torsion angles; correlations between esds in cell parameters are only used when they are defined by crystal symmetry. An approximate (isotropic) treatment of cell esds is used for estimating esds involving l.s. planes.
Refinement. Refinement of *F*^2^ against ALL reflections. The weighted *R*-factor wR and goodness of fit *S* are based on *F*^2^, conventional *R*-factors *R* are based on *F*, with *F* set to zero for negative *F*^2^. The threshold expression of *F*^2^ > σ(*F*^2^) is used only for calculating *R*-factors(gt) etc. and is not relevant to the choice of reflections for refinement. *R*-factors based on *F*^2^ are statistically about twice as large as those based on *F*, and *R*- factors based on ALL data will be even larger.

### Fractional atomic coordinates and isotropic or equivalent isotropic displacement parameters (Å^2^)

**Table:**

	*x*	*y*	*z*	*U*_iso_*/*U*_eq_	Occ. (<1)
S1	0.44974 (10)	0.67774 (2)	0.55925 (11)	0.03155 (18)	0.839 (2)
C5A	0.446 (2)	0.6612 (2)	0.5331 (18)	0.0246 (5)	0.161 (2)
H5A	0.4003	0.7061	0.4718	0.030*	0.161 (2)
C2	0.4207 (2)	0.59508 (6)	0.4154 (2)	0.0255 (3)	
H2	0.3574	0.5891	0.2673	0.031*	
C3	0.50025 (17)	0.53842 (7)	0.5407 (2)	0.0199 (3)	
C4	0.58337 (19)	0.56308 (7)	0.7572 (3)	0.0262 (3)	
H4	0.6439	0.5314	0.8666	0.031*	
C5	0.5674 (8)	0.63775 (10)	0.7926 (7)	0.0246 (5)	0.839 (2)
H5	0.6150	0.6636	0.9277	0.030*	0.839 (2)
S1A	0.5658 (13)	0.65626 (13)	0.7988 (13)	0.0391 (12)	0.161 (2)

### Atomic displacement parameters (Å^2^)

**Table:**

	*U*^11^	*U*^22^	*U*^33^	*U*^12^	*U*^13^	*U*^23^
S1	0.0353 (3)	0.0217 (2)	0.0377 (3)	0.0028 (2)	0.0068 (2)	0.00256 (18)
C5A	0.0243 (10)	0.0215 (10)	0.0279 (10)	−0.0028 (13)	0.0000 (8)	−0.0033 (10)
C2	0.0264 (7)	0.0274 (6)	0.0227 (6)	0.0032 (5)	0.0010 (5)	0.0039 (5)
C3	0.0152 (6)	0.0247 (6)	0.0198 (6)	0.0022 (5)	0.0025 (5)	0.0022 (5)
C4	0.0234 (7)	0.0303 (7)	0.0249 (6)	0.0026 (5)	−0.0034 (5)	−0.0015 (5)
C5	0.0243 (10)	0.0215 (10)	0.0279 (10)	−0.0028 (13)	0.0000 (8)	−0.0033 (10)
S1A	0.040 (2)	0.0276 (17)	0.049 (2)	−0.001 (2)	0.0038 (15)	−0.0074 (17)

### Geometric parameters (Å, °)

**Table:**

S1—C2	1.7152 (9)	C3—C4	1.4181 (19)
S1—C5	1.7215 (10)	C3—C3^i^	1.470 (3)
C5A—C2	1.3802 (10)	C4—C5	1.3794 (10)
C5A—S1A	1.7203 (10)	C4—S1A	1.7180 (10)
C5A—H5A	0.9500	C4—H4	0.9500
C2—C3	1.3778 (19)	C5—H5	0.9500
C2—H2	0.9500		
			
C2—S1—C5	92.19 (8)	C4—C3—C3^i^	123.98 (15)
C2—C5A—S1A	115.2 (3)	C5—C4—C3	113.15 (13)
C2—C5A—H5A	122.4	C3—C4—S1A	113.04 (17)
S1A—C5A—H5A	122.4	C5—C4—H4	123.4
C3—C2—C5A	111.1 (2)	C3—C4—H4	123.4
C3—C2—S1	111.81 (10)	S1A—C4—H4	123.5
C3—C2—H2	124.1	C4—C5—S1	110.87 (12)
C5A—C2—H2	124.9	C4—C5—H5	124.6
S1—C2—H2	124.1	S1—C5—H5	124.6
C2—C3—C4	111.98 (11)	C4—S1A—C5A	88.8 (2)
C2—C3—C3^i^	124.05 (15)		
			
S1A—C5A—C2—C3	−0.2 (15)	C3^i^—C3—C4—C5	179.3 (3)
C5—S1—C2—C3	−0.6 (3)	C2—C3—C4—S1A	−1.3 (5)
C5A—C2—C3—C4	1.0 (8)	C3^i^—C3—C4—S1A	178.5 (4)
S1—C2—C3—C4	0.73 (16)	C3—C4—C5—S1	0.1 (5)
C5A—C2—C3—C3^i^	−178.8 (8)	C2—S1—C5—C4	0.3 (4)
S1—C2—C3—C3^i^	−179.03 (14)	C3—C4—S1A—C5A	1.0 (10)
C2—C3—C4—C5	−0.5 (3)	C2—C5A—S1A—C4	−0.4 (14)

Symmetry codes: (i) −*x*+1, −*y*+1, −*z*+1.

### Hydrogen-bond geometry (Å, °)

**Table:**

Cg and Cg' are the centroids of the thiophene ring in the major and minor occupancy disorder components, respectively.

**Table:**

*D*—H···*A*	*D*—H	H···*A*	*D*···*A*	*D*—H···*A*
C2—H2···Cg^ii^	0.95	2.86	3.6039 (17)	136
C2—H2···Cg'^ii^	0.95	2.86	3.607 (5)	136

Symmetry codes: (ii) −*x*+1/2, *y*, *z*−1/2.
